# Small Cell Carcinoma of Prostate: A Case Report of a Patient With Concomitant Transitional Cell Cancer of the Bladder

**DOI:** 10.1177/2324709618760644

**Published:** 2018-03-05

**Authors:** Waiel Abusnina, Eric Yiman Auyoung, Mohammed Megri, Toni Pacioles

**Affiliations:** 1 Department of Internal Medicine, Joan C. Edwards School of Medicine, Marshall University, USA; 2 Department of Hematology/Oncology, Joan C. Edwards School of Medicine, Marshall University, USA

**Keywords:** small cell cancer, prostate cancer

## Abstract

Small cell carcinomas (SCCs) are aggressive neoplasms commonly associated with a pulmonary origin. However, albeit rare, extrapulmonary SCC can occur in a variety of sites with an incidence in North America approximated to be 0.1% to 0.4%. Among these sites, approximately 10% of extrapulmonary SCC cases occur in the prostate and are associated with a poor mortality with a median survival of 10 months. Because of the rarity of the prostatic SCC, there is no formal treatment protocol. In this case report, we present a patient who was diagnosed with SCC in the prostate as primary origin. Adjuvant concurrent chemoradiotherapy was started, which he is tolerating so far. While the management of metastatic disease is well documented with the use of chemotherapy, specific data on nonmetastatic disease is lacking. As some studies suggest, a combined surgical and chemotherapeutic approach is helpful in localized disease. In our case, this approach has led to a good clinical outcome in a disease that does not usually allow such results.

## Introduction

Small cell carcinomas (SCCs) are aggressive neoplasms commonly associated with a pulmonary origin. However, albeit rare, extrapulmonary SCC (EPSCC) can occur in a variety of sites with an incidence in North America approximated to be 0.1% to 0.4%.^1^ Among these sites, approximately 10% of EPSCC cases occur in the prostate^2,3^ and are associated with a poor survival, with a median survival of 10 months.^3,4^ Because of the rarity of the prostatic SCC, there is no formal treatment protocol. Current treatment is based on studies of pulmonary SCC, using a combined chemoradiotherapeutic approach with radical prostatectomy as an adjunct in only a few selected cases.^5-8^ With the poor outcome of this aggressive subtype of prostate cancer, further studies can help improve treatment outcomes. Here, we present a case of limited-stage pure SCC of the prostate treated with a total cystoprostatectomy and adjuvant chemoradiotherapy.

## Case Report

**Figure 1.**
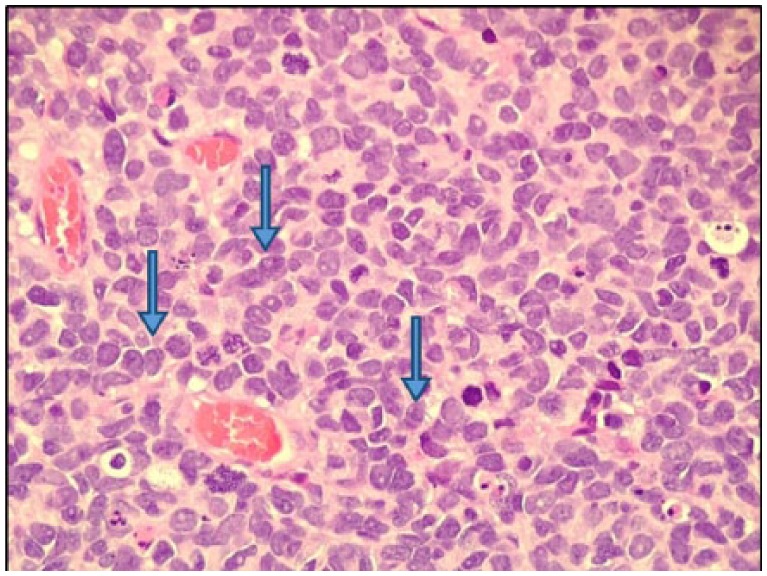
The tumor is composed of cells with hyperchromatic nuclei, no nucleoli, scant cytoplasm, and nuclear molding, with numerous mitoses (hematoxylin-eosin, 400×).

**Figure 2.**
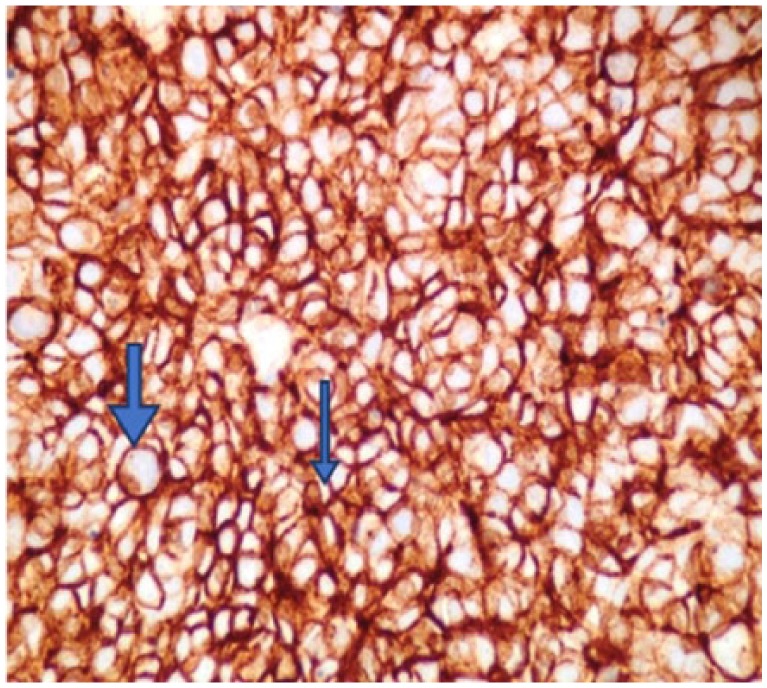
The tumor has neuroendocrine features as shown by positivity with antibodies to CD56 (400×).

**Figure 3.**
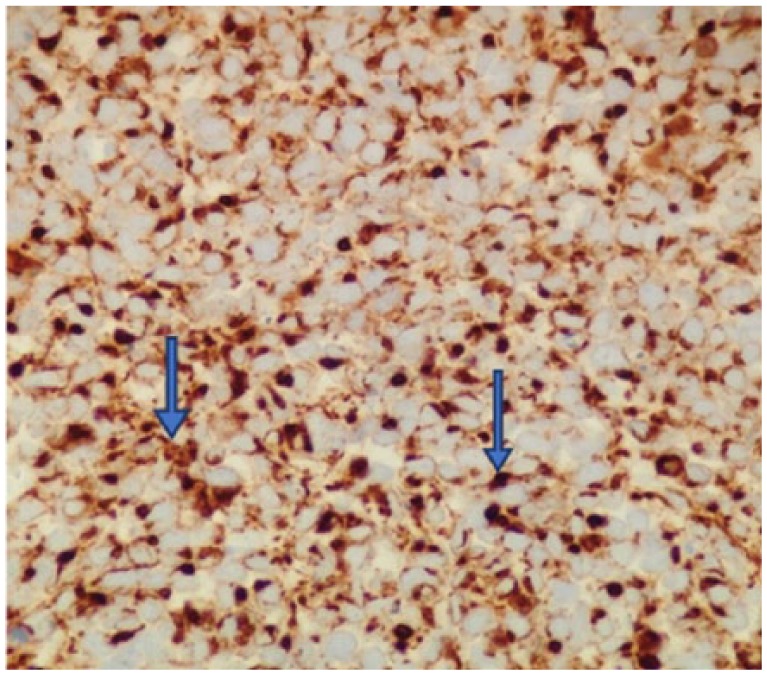
The tumor has neuroendocrine features as shown by positivity with antibodies to synaptophysin (400×).

A 60-year-old Caucasian male presented with episodes of urinary retention, a normal digital rectal examination, and no other urinary symptoms. His past medical history was significant for a high-grade T1 transitional cell carcinoma of the bladder 1 year prior, as well as another T1 transitional cell carcinoma of the bladder 7 months after the first tumor was diagnosed. For the first tumor, he successfully underwent transurethral resection of the tumor and 6 cycles of intravesical bacillus Calmette-Guerin (BCG) immunotherapy. The same treatment plan was given for his second tumor, but he developed BCG-osis after the fourth cycle. He underwent close follow-up by his urologist with abdominal/pelvic computed tomography (CT) scans and urine cytology. Three months later, around the onset of his urinary retention, imaging showed thickening of his bladder wall, and urine cytology was positive for malignancy. Furthermore, on bimanual examination, a firm, but moveable mass was palpated. With these new findings, the patient was recommended cystoscopy with transurethral resection of the prostate to investigate a suspected recurrence of bladder cancer and to provide relief of his obstructive symptoms. The procedure relieved not only the patient’s urinary retention but also revealed a fleshy mass occupying the right lobe of the prostate and no bladder masses. The pathologist reported the transrectal ultrasound-guided prostate biopsy to be consistent with a small cell neuroendocrine carcinoma. High-power evaluation (400×) demonstrated tumor cells with hyperchromatic nuclei, no nucleoli, scant cytoplasm, and nuclear molding, with numerous mitoses (Figure 1). The tumor has neuroendocrine features as shown by positivity with antibodies to CD5 (Figure 2) and synaptophysin (Figure 3).

**Figure 4.**
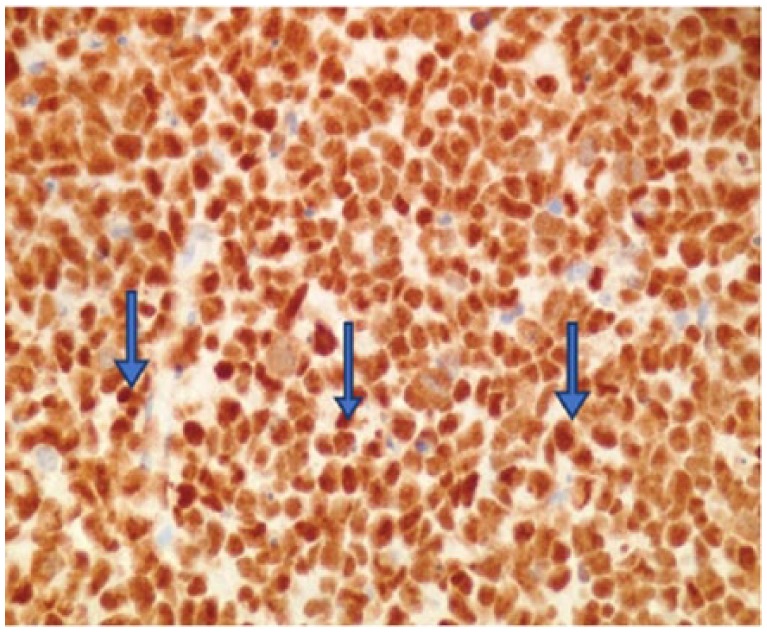
p53 mutation is shown by positive nuclear staining with antibody to p53 (400×).

**Figure 5.**
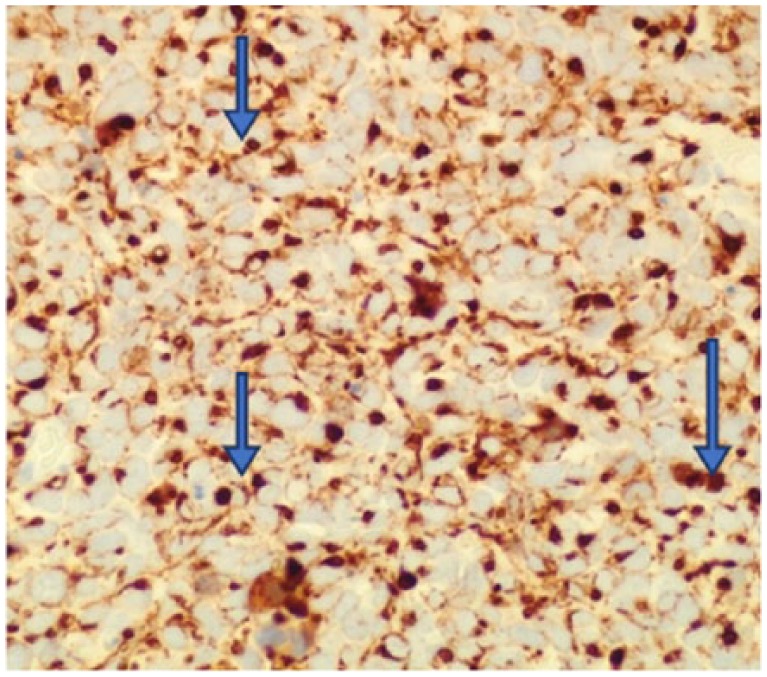
The tumor is a carcinoma as evidenced by positivity with antibodies to CK7 (400×).

**Figure 6.**
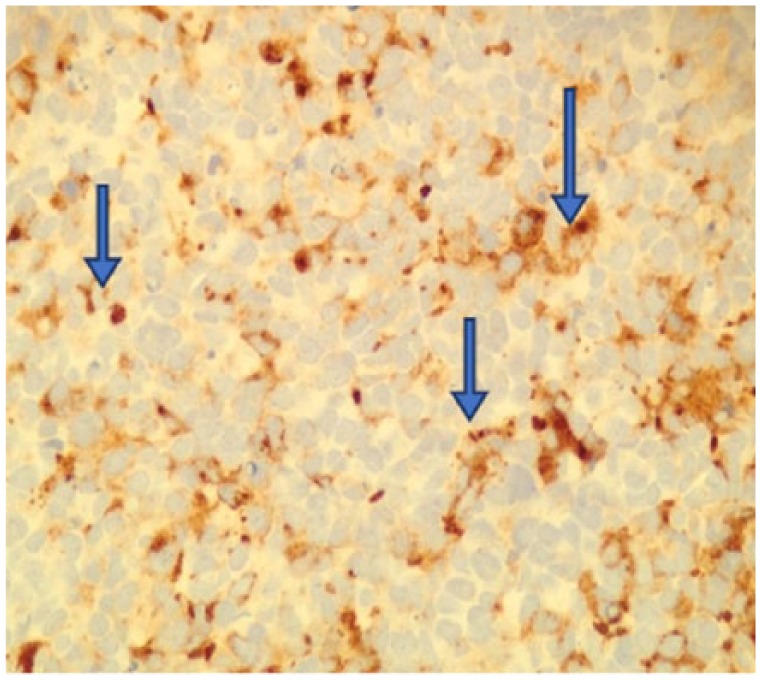
The tumor is a carcinoma as evidenced by positivity with antibodies to cytokeratin AE1/AE3 (400×).

Immunohistochemical stains were found to be positive for P53, CK7, and AE1/AE3 (Figures 4-6) but negative for staining with antibodies to chromogranin A, CK20, PSA, uroplakin III, GATA-3, and LCA (data not shown).

The specimen was also compared with the patient’s previous bladder biopsy and determined to be morphologically dissimilar. Further staging workup was obtained with positron emission tomography (PET)/CT scan and with magnetic resonance imaging of the pelvis–neither of which revealed obvious lymphadenopathy or extraprostatic spread. Considering the localized nature of the tumor, the patient underwent a robotic-assisted total cyst prostatectomy with lymph node sampling of the right and left obturator lymph nodes. Final pathology report confirmed a submucosal small cell neuroendocrine carcinoma in the prostatic base, 3.5 cm at its greatest dimension, with involvement of the bladder neck and trigone area. One of the 2 lymph nodes were positive for metastatic carcinoma, and final pathologic staging was determined to be T3aN1M0. He underwent adjuvant concurrent chemoradiotherapy with cisplatin/etoposide. The patient completed 4 cycles and at last follow-up, 12 months after diagnosis, is tolerating the treatment well with negative CT imaging for recurrence.

## Discussion

Although the prostate is one of the common site for EPSCC, it is a rare prostatic malignancy.^2,3,9^ Of these cases, only about 35% are pure SCC of the prostate, with the rest having concurrent adenocarcinomatous components,^5^ but it is currently unknown whether this pathological difference affects the management or prognosis. SCC of the prostate is reported to be more prevalent in the elderly with over 70% of patients being over the age of 65.^10^ Unlike prostate adenocarcinomas, most patients with SCC of the prostate are symptomatic. The most frequently reported symptoms are obstructive symptoms, pressure-related neurological symptoms, or constitutional symptoms, with 10% of cases present with paraneoplastic symptoms.^9,11^ Depending on the tumor involvement, other clinical features include bone pain, hydronephrosis, abdominal pain, hematochezia, and hematuria. Imaging of the pelvis is generally nonspecific and serum prostate-specific antigen levels often remain within normal ranges.^5^ Ultimately, SCC of the prostate is diagnosed by pathological analysis of morphology and immunohistochemistry.^3,5,11^

Much of our current treatment approach to prostatic SCC are extrapolated from studies on the management of pulmonary SCC. In the limited studies available on the management of prostatic SCC, the recommended first-line treatment is chemotherapy with a combined use of cisplatin and etoposide because of its comparatively higher efficacy rate.^5,6,11^ However, most of these cases are patients with metastatic or extensive-stage prostatic SCC, the more common presentation of the tumor. With respect to nonmetastatic or limited-stage prostatic SCC, a couple of studies recommend prostatectomy with adjuvant chemotherapy, but this approach is controversial.^2,5,12^ Most studies report using chemotherapy as the first-line treatment regardless if the tumor is metastatic or not; hence, it is difficult to properly assess the efficacy of a surgical therapy with adjuvant chemotherapy. Several studies have reported that prostatectomy alone is not effective.^5,8,13^ In these localized tumors, as in our case, it would be logical to remove the tumor to potentially prevent possible metastases and use adjuvant chemotherapy to treat possible occult metastases.

## Conclusion

SCC of the prostate is an aggressive tumor that is associated with a high mortality. While the management of metastatic disease is well documented with the use of chemotherapy, specific data on nonmetastatic disease are lacking. As some studies suggest, a combined surgical and chemotherapeutic approach is helpful in localized disease. In our case, this approach has led to a good clinical outcome in a disease that does not usually allow such results.
